# Sleep and Health in Older Adults: New Research From Early-Career Investigators

**DOI:** 10.1093/geroni/igab046.126

**Published:** 2021-12-17

**Authors:** Christopher Kaufmann, Amy Berkley

## Abstract

Sleep and circadian patterns change as people age and are linked to a number of health and psychosocial outcomes. As such, there is a need to continue generating new knowledge about sleep in older adults by encouraging early-career scientists to research this topic. In this symposium, sponsored by the Sleep, Circadian Rhythms and Aging Interest Group, we will showcase studies by early-career researchers at the masters through junior faculty level who conduct work in sleep and its impact on health outcomes in older adults. Our symposium will have five presentations. The first will examine how sleep and loneliness may mediate relationships between marital quality and depressive symptoms. The second study will assess links between personality characteristics and objectively measured chronotype. Our third presentation will determine the longitudinal association of sleep duration with body mass index. The fourth will evaluate how an intervention to reduce functional disability in low-income older adults impacts sleep quality. Finally, our fifth presentation will focus on understanding how sleep duration and changes in sleep patterns may impact speech-in-noise performance. Overall, our symposium will highlight multidisciplinary studies of sleep and health outcomes that are of importance to older populations and promote the work of the next generation of sleep, circadian rhythms, and aging scientists.

